# How Smart are Smart Materials? A Conceptual and Ethical Analysis of Smart Lifelike Materials for the Design of Regenerative Valve Implants

**DOI:** 10.1007/s11948-023-00453-1

**Published:** 2023-09-05

**Authors:** Anne-Floor J. de Kanter, Karin R. Jongsma, Carlijn V. C. Bouten, Annelien L. Bredenoord

**Affiliations:** 1 Department of Bioethics and Health Humanities, Julius Center for Health Sciences and Primary Care, University Medical Center Utrecht, Utrecht University, 3508 GA Utrecht, The Netherlands; 2 Department of Biomedical Engineering and Institute for Complex Molecular Systems, Eindhoven University of Technology, 5600 MB Eindhoven, The Netherlands; 3 Erasmus School of Philosophy, Erasmus University Rotterdam, 3062 PA Rotterdam, The Netherlands

**Keywords:** Regenerative medicine, Implantable devices, Smart materials, Biomimicry, Irreversibility, Embodiment, Entanglement

## Abstract

It may soon become possible not just to replace, but to re-grow healthy tissues after injury or disease, because of innovations in the field of Regenerative Medicine. One particularly promising innovation is a regenerative valve implant to treat people with heart valve disease. These implants are fabricated from so-called ‘smart’, ‘lifelike’ materials. Implanted inside a heart, these implants stimulate re-growth of a healthy, living heart valve. While the technological development advances, the ethical implications of this new technology are still unclear and a clear conceptual understanding of the notions ‘smart' and ‘lifelike' is currently lacking. In this paper, we explore the conceptual and ethical implications of the development of smart lifelike materials for the design of regenerative implants, by analysing heart valve implants as a showcase. In our conceptual analysis, we show that the materials are considered ‘smart’ because they can communicate with human tissues, and ‘lifelike’ because they are structurally similar to these tissues. This shows that regenerative valve implants become intimately integrated in the living tissues of the human body. As such, they manifest the ontological entanglement of body and technology. In our ethical analysis, we argue this is ethically significant in at least two ways: It exacerbates the irreversibility of the implantation procedure, and it might affect the embodied experience of the implant recipient. With our conceptual and ethical analysis, we aim to contribute to responsible development of smart lifelike materials and regenerative implants.

## Introduction

Modern transplantation medicine is vastly influenced by technological interventions. For example, a hip implant can replace a diseased, non-functional hip joint; a pacemaker can sustain the beating rhythm of a human heart. While these interventions sustain and restore tissue and organ function, the human body cannot re-grow a living tissue or organ—like a hip bone or a heart valve. Regenerative Medicine is the field of study that is looking for ways to grow or engineer living human tissues and organs in order to regenerate parts of the human body after injury or disease.

One particularly promising Regenerative Medicine innovation—that we use as a showcase in this paper—is a regenerative valve implant for people with heart valve disease (Bouten et al., 2012, 2018; Wissing et al., 2017), as developed in the Dutch Materials-driven Regeneration programme (see Box 1.) and elsewhere. What is special about this implant is that it is made of a synthetic material^1^ that is designed to be slowly broken down by the body, while the body grows new valve tissue in its place. The implant material thus acts as a temporary cell scaffold. Over time, the body will regenerate a healthy living heart valve.

**Box 1:** Materials-driven Regeneration (MDR)

This paper results from a research project on the ethical implications of Regenerative Medicine that is conducted as part of the Materials-driven Regeneration (MDR) programme, a 10-year research programme funded by the Dutch Gravitation funding scheme from the Dutch Research Council. This programme brings together scientists from chemistry, bioengineering, and biofabrication with cardiologists, nephrologists, and orthopaedic surgeons in a joint effort to develop new regenerative therapies for the heart, kidney and skeletal system based on smart, lifelike materials. The development of regenerative heart valve implants is a major focus point of MDR that is taken as the showcase of this paper. The findings presented in this paper are partly informed by our position as ethicists (AK, KJ, AB) embedded in the MDR programme	

The promise of regenerative valve implants is that they will provide a life-long cure and will become an off-the-shelf available and cost-effective treatment, particularly suitable for children (Bouten et al., 2018; Fioretta et al., 2021). As such, the implants promise to circumvent complications and limitations of current treatment options using mechanical or biological implants.^2^ Currently, proof-of-principle for this approach has been provided in sheep (Kluin et al., 2017), and the first small-scale clinical trial has been initiated (XPlore-II by Xeltis BV, NCT03022708).

Regenerative valve implants are fabricated out of a so-called smart lifelike material (see Box 2.) (Bouten et al., 2012; Wu et al., 2017). This material has been designated ‘smart’ and ‘lifelike’ because, the implants derive their capacity to stimulate tissue regeneration from the properties of this material. In the literature, this approach to tissue regeneration is referred as in situ tissue engineering (Abdulghani & Mitchell, 2019; Bouten et al., 2018). This differs from ‘conventional’ tissue engineering approaches that rely largely on the presence of (stem) cells on the implant construct prior to implantation (Hoerstrup et al., 2006). In addition to heart valve regeneration, in situ tissue engineering is also being explored for regeneration of vessels (Li et al., 2014), bone (Cao et al., 2020; Vermeulen et al., 2022; Wei et al., 2022), and cartilage in joints (Wang et al., 2022).

**Box 2:** Technological background: supramolecular materials

The smart lifelike materials developed by the MDR programme (see Box 1) are so-called supramolecular materials. Supramolecular materials are polymers with repetitive binding units that cluster together to form versatile superstructures. Chemists can tweak these superstructures with desirable properties by adding chemical units with known bioactive behaviours. These bioactive units can transmit signals to cells in living tissues through molecular signalling pathways. Chemical engineers discover these units intentionally—and smartly—by trial-and-error or by rational design. That way, chemists can endow materials with ‘smart’ and ‘lifelike’ properties to design implants that stimulate regeneration of tissue in a human body	

While the technological development advances, the ethical implications of this new technology are still unclear. The ethical aspects of Regenerative Medicine as a field, and stem cell developments in particular, have gained ample attention over the last decade (Assen et al., 2021; Chan, 2017; Cossu et al., 2018; De Kanter et al., 2023; Hyun, 2010). Yet the potential ethical and societal implications of scaffold materials and in situ tissue engineering have only recently become a topic of interest (Hunckler & Levine, 2022; Parry, 2018; Van Daal, De Graeff, De Kanter, Bredenoord, manuscript submitted for publication). Elucidating the ethical aspects of smart lifelike materials and regenerative implants requires a clear conceptual understanding of the notions ‘smart’ and ‘lifelike’, which is currently lacking.

In this paper, we explore the conceptual and ethical implications of the development of smart lifelike materials for the design of regenerative implants, by analysing heart valve implants as a showcase for regenerative implants. In our conceptual analysis, we clarify how the notions ‘smart’ and ‘lifelike’ are used and understood in the context of material development. Next, we discuss what these insights reveal about the ontological status of regenerative valve implants. The regenerative valve implants become intimately integrated in the living tissues of the human body, and as such, they manifest the ontological entanglement of body and technology. In our ethical analysis, we argue that this is ethically significant in at least two ways: It exacerbates the irreversibility of the implantation procedure, and it might affect the embodied experience of the implant recipient. In our discussion, we explore what new forms of smartness and lifelikeness might be developed in the future, and how this may change the design of regenerative implants. With our analysis, we aim to foster responsible development of smart lifelike materials and regenerative implants.

## Conceptual Analysis of Smart Lifelike Materials

The meaning of ‘smart’ and ‘lifelike’ is not self-evident, and no widely shared definition of either has been provided in the scientific literature. In the following, we conceptualise the notions ‘smart’ and ‘lifelike’, based on how the notions are presented in scientific and philosophical literature (see also Table 1). We will focus in particular on the use of these notions in the context of materials for Regenerative Medicine.

**Table 1:** We propose the following understanding of the notions ‘smart’ and ‘lifelike’, in the context of Regenerative Medicine and in the context of other technologies.

	Smart	Lifelike
Regenerative Medicine	Smart = the two-way communication (sending signals vs. receiving and adapting to signals) of materials with the tissues of the body through molecular signalling pathways *Context: smart materials in Regenerative Medicine*	Lifelike = the structural and functional similarity of material to, integration in, and interaction with the environment of living cells *Context: lifelike materials in Regenerative Medicine*
Other technologies	Smart = the collection and exchange of digital or electronic data *Context: smart information technologies (*Haddow et al., 2016*)*	Biomimicry = the technological differentiation from a natural phenomenon that remains embedded and participates in the natural environment *Context: biomimetic technologies in ecological design (*Blok, 2023*)*

### Smartness

‘Smart’ as a description of materials can frequently be found in scientific literature in the domain of chemical and material sciences (Bouten et al., 2012; Raman & Bashir, 2017). ‘Intelligent’ is sometimes used as a synonym (Bensaude-Vincent et al., 2002; Liu et al., 2020). In principle, ‘smart’ could be understood to refer to the engineer and the engineering process, or to the material itself. While materials are intentionally—and smartly—designed by chemical engineers, ‘smart’ is usually presented in the literature as a characteristic of the material itself, in two distinct ways.

First, ‘smart’ refers to the ability of materials to sense and respond to changes in their environment. For example, certain materials can shrink or expand in response to acidification (pH) or solidify in response to temperature or light (Jingcheng et al., 2021; Kowalski et al., 2018; Raman & Bashir, 2017; Tian et al., 2022)—a property that is also exploited in 3D biofabrication technologies. ‘Smart’ in this sense can also refer to the mode and speed of material degradation, which for regenerative valve implants should closely match the pace of new tissue growth (Brouns & Dankers, 2021). Second, particularly in the context of regenerative valve implants, ‘smart’ refers to the ability of materials to attract cells from the body (e.g. immune cells) to the implant environment and to guide subsequent tissue regeneration using physical or chemical cues (Blacklow et al., 2019; Lei et al., 2021; Ooi et al., 2017; Sands & Mooney, 2007).

‘Smartness’ thus refers to the ability of the implants to ‘communicate’ back and forth, i.e. to send and receive signals, with the cells of the surrounding tissue. In the forward direction, the materials stimulate the living tissue to start the process of regeneration. In the backward direction, the synthetic materials respond by adapting (e.g. shrinking or decomposing) their physical structure as needed.

### Lifelikeness

‘Lifelike’ is another descriptor of the materials out of which regenerative valve implants are made (Wu et al., 2017). Similar (but not synonymous) to lifelike, the literature also refers to these materials as ‘biomimetic’ (Bensaude-Vincent et al., 2002; Raman & Bashir, 2017). *Biomimicry* is an influential design principle for taking inspiration from and learning from nature, not only in the context of Regenerative Medicine, but also in ecological and sustainable design, where the term originates (Benyus, 2009). Biomimicry (as a form of *mimesis*) can be defined as differentiation of a technological design from a natural phenomenon, that however remains embedded and participates in the natural environment (Blok, 2023). As such, biomimicry differs from mere (undifferentiated) imitation of nature on the one hand and from bioinspiration as nature-inspired invention of something completely new (and non-embedded) on the other (Blok, 2023).

Lifelikeness in the context of regenerative valve implants can be understood in a similar manner. Lifelike materials have been designed to mimic the so-called extracellular matrix, which is the scaffold structure that naturally accommodates living cells. Moreover, lifelike materials become embedded in the living environment of the human body, i.e. they are designed to be seeded with living cells, or in case of regenerative valve implants, designed to be implanted *within* living tissues. Yet, lifelikeness does not refer to the mere ability to serve as a cell scaffold—a property lifelike materials share with many other biocompatible materials and hydrogels (i.e. with *bio*materials^3^ in general). Instead, as a form of biomimicry, lifelikeness refers specifically to the attempt to mimic closely the protein and sugar configurations of extracellular matrices which play an essential role in molecular signalling pathways and communication between cells. As such, lifelike materials are not only structurally and functionally similar to living systems but are also able to interact with them and thus participate in the living environment of the human body (a feature on which the ‘smart’ properties also rely, as described).

Thus, while the adjectives ‘lifelike’ or ‘biomimetic’ imply a similarity to living systems, lifelikeness does not entail near-living characteristics of the material itself (e.g. the materials cannot self-reproduce). Instead, it refers to the molecular architecture and the physical, chemical and functional properties of the materials, which are similar to that of the living systems in which they are embedded and with which they communicate. Because of this similarity and embeddedness, lifelike materials can act as a host for living cells, inviting them to migrate into the material, to make themselves ‘at home’ and proliferate.

## Ontological Status of Regenerative Valve Implants

This conceptual understanding of smart lifelike materials provides insights into the ontological status of regenerative valve implants.

We showed above that the materials are considered ‘smart’ because they can ‘communicate’ with human tissues, and ‘lifelike’ because they are structurally and functionally similar to and embedded in these tissues. Together, this fosters a process of dynamic interaction through which the lifelike fabrics of the synthetic material integrate intimately with the living tissues of the human body. Cells grow into the material, the material dissolves into the body and over time the implant is transformed into a living tissue. It is at this physical and functional continuity between implant and body that the regenerative potential of the valve implants is realised.

As such, regenerative valve implants manifest an increasing entanglement of technological artefacts with the human body. The implants start out as technological artefacts (Vermaas et al., 2011), non-living physical entities created by humans for a human purpose. Yet after entering the intimate and private sphere of the human body, they are transformed into living tissues and become one with this body, that is both object and subject.

Regenerative valve implants differ from traditional prosthesis and inert implants because their physicality and action can no longer be easily and clearly discerned from the human body in which they are implanted (Parry, 2018). In contrast to other types of implantable devices that also perform or sustain bodily functions, such as ventricular assist devices or pacemakers, regenerative valve implants become physically interwoven with (and finally disintegrate and dissolve into) the body, and the entities of body and technology become immersed or *entangled* on an ontological level too. This challenges existing ontological distinctions between the categories of human and technology, body and device. It blurs the boundaries between the technological and the corporeal (cf. Parry, 2018), between natural and artificial (Bensaude-Vincent et al., 2017), between life and non-life (Webster, 2016).

Notably, the entanglement of the regenerative valve implants with the living body is directly indicated by the smartness and lifelikeness of the materials of which the implants are made. Smartness emphasises the communication, dynamic interaction and mutual responsiveness between the synthetic material and the living tissues. Lifelikeness emphasises the similarity of the materials to aspects of the living tissues, as well as their active embeddedness and integration into these tissues. All of these add to the entanglement between synthetic material and living tissue, between technology and body.

## Ethical Analysis of Regenerative Valve Implants

The entanglement of the technological and corporeal—as indicated by the notions ‘smart' and ‘lifelike'—is ethically significant in at least two ways: It may exacerbate the irreversibility of the implantation procedure and it may impact the embodied experience of the recipients of regenerative valve implants, as we explain below.

### Irreversibility

The integration of regenerative valve implants in the tissues of the body adds a highly *irreversible* dimension to the implants (Trommelmans et al., 2008, 2009; Van Daal, De Graeff, De Kanter, Bredenoord, manuscript submitted for publication). Implant degradation and tissue regeneration are irreversible processes. Moreover, once cellular ingrowth or degradation of the material itself has started, it might be complex, if not impossible, to remove the implant. Eventually, the implant can no longer be distinguished from living tissue and the surgical implantation procedure itself becomes irreversible. This could become problematic, especially if medical complications arise that are harmful for the recipient, as we discuss below.

The irreversibility of regenerative valve implants has implications for *risk* and *responsibility*. We will first illustrate this through analogy with a recent case study on a spinal cord implant for the treatment of lower back pain (Parry, 2018), and then return to regenerative valve implants. Like regenerative valve implants, the spinal cord implant was designed to stimulate tissue regeneration. In contrast to regenerative valve implants, the spinal cord implant is not made of a single material but is an assembly of different components: it consists of a hollow metal casing filled with a scaffold material and loaded with growth factors (Parry, 2018). While the metal casing acts as a traditional inert implant, the scaffold material together with the growth factors interacts with the surrounding tissues and stimulate bone regeneration.

As regards *risk*, the spinal cord implant, while initially promising, turned out to cause serious complications in many recipients. This included damage to the airways and reproductive organs (including swelling of the throat tissues and male sterility) and increased risk of cancer (Parry, 2018). Moreover, removal of the implant involved additional risks, while if the implant would or could not be removed, this meant that recipients had to forego the opportunity of another better treatment. Irreversibility of the implantation procedure thus increased the risks involved.

Irreversibility also complicates the question who should be held *responsible* when complications arise. Again, this can be illustrated by analogy with the spinal cord implant. The company manufacturing the implant lobbied for the implant to be regulated as a medical device (Parry, 2018). Devices are subjected to less strict safety assessment than drugs, because their therapeutic effect is supposed to be reversible by removal of the device. This assumption of reversibility turned out to be erroneous for the spinal cord implant: the growth factors, that were supposed to be therapeutically active in the implantation area only, migrated throughout the body causing major complications (Parry, 2018). The biological activity of the implant got out of control. Yet the manufacturing company evaded liability for most claims by dismissing adverse events as ‘excessive bodily responses’, shifting the blame to the implant recipients and their unpredictable bodies instead (Parry, 2018). Thus, because of an erroneous assumption on the reversibility of the spinal cord implant, an—arguably unjust—shift took place in to whom responsibility for complications was allocated.

For smart lifelike materials and regenerative valve implants, irreversibility is exacerbated compared to the spinal cord implant. While the metal casing of the spinal cord implant is inert and remains a clearly circumscribed entity that can be discerned from the corporeal environment (also long after it has been implanted), regenerative valve implants are explicitly designed to enter into a dynamic interaction with the tissues of the body, with which, over time, they will become one. Slowly, the physical boundaries between regenerative valve implants and the body will disappear. The processes of implant degradation and cellular ingrowth are essential aspects of the therapeutic mode-of-action of regenerative valve implants but are also inherently irreversible. In other words, for regenerative valve implants irreversibility is inherent to the implant design.

Irreversibility and disintegration of regenerative valve implants might change the *risks* involved—or bear still unknown risks—as compared to standard surgical interventions and heart valve implants. It might be impossible to remove a regenerative valve implant in its entirety, or to do so without damaging the emerging and surrounding tissue. And even if possible, removing the partly degraded and remodelled implant might be insufficient to reverse harmful processes if complications arise, e.g. in case of potential toxicity of degradation products, or unforeseen or uncontrollable tissue growth and adaptation (Simionescu et al., 2011; Uiterwijk et al., 2020). Other risks include disbalance between the pace of tissue growth and implant degradation (Duijvelshoff et al., 2021), and the risk of implant calcification (Van der Valk et al., 2023). The degree of risk compared to existing valve implants or other surgical interventions is currently unknown. Treatment outcomes might differ between people, which adds additional uncertainty (De Kort et al., 2021).

Finally, irreversibility and disintegration of regenerative valve implants also have implications for *responsibility*, particularly in relation to loss of (human) control. By offering the implants in their smart, lifelike form to the body, engineers aim to steer or control bodily processes in order to stimulate the body to regenerate itself. Because this approach relies on the inherent regenerative capacity of the human body, the degree of control that engineers have over the process of regeneration is overall limited.^4^ While (bio)engineering is a discipline that normally aims to control natural processes, in case of regenerative implants, the engineer must relinquish control and trust that the body will take over and regenerate itself. This leaves the engineer, as well as the physician and recipient, at mercy of the dynamic processes taking place in the interaction between implant and body (Haddow et al., 2016; Oerlemans et al., 2013). As the case study on the spinal cord implant showed, the unpredictability and irreversibility of bodily processes can pose challenges for the allocation of responsibility for treatment outcomes. Letting go of control might mean that responsibility becomes distributed over different parties who are part of the chain of development and care.

Overall, the irreversibility inherent to the design of regenerative valve implants adds to the risks involved and complicates the allocation of responsibility.

### Embodiment

Living with an implant might affect the way in which the implant recipient experiences their body and relates to their body in everyday life. That is, living with an implant might affect the *embodied* experience of the recipient. We argue that the physical integration between regenerative valve implants and living tissues, and the entanglement between technology and body, might matter to the process of incorporating a regenerative implant, which could affect embodiment of the recipient, as will be substantiated below.

*Embodiment* is a notion from the phenomenological tradition that refers to the status of the body as both object (as part of the physical world) and subject (as our means to perceiving and experiencing the world) (Moore et al., 2022; Slatman, 2014). In a neutral state of embodiment, the body and self are lived as one (Lape et al., 2019). When this unity is disrupted, one might become unpleasantly aware of their body (hyperembodiment), or one may feel alienated towards their body (disembodiment) (Lape et al., 2019). *Incorporation* refers to the process of integrating something (an object or a habit) into the body, so that it is experienced as part of the body as it is lived in everyday life (Colombetti, 2016; Moore et al., 2022; Slatman, 2014). Incorporation of an implant is not a straightforward result of the implantation procedure but is rather an active process of (re-)identification with one’s body, done by the recipient in relation with others (loved ones) (Dalibert, 2016).

For people living with an implant, successful embodiment and incorporation matter especially for the therapeutic success of the treatment (Dalibert, 2016; Lape et al., 2019). We will first illustrate this by discussing a recent case study on knee implants, and then discuss the role of embodiment and incorporation in living with regenerative valve implants, which we hypothesise is affected by the physical integration between the implant and the tissues of the body.

Two recent studies have explored the role of embodied experience to explain recipients’ dissatisfaction with knee implants (Lape et al., 2019; Moore et al., 2022). Knee implants are usually successful for the treatment of osteoarthritis. For most recipients, knee implants restore daily activities, diminish alienation caused by a period of illness and improve everyday lived experience. Unfortunately, some recipients remain dissatisfied and experience chronic pain. Empirical research shows that for (at least part of) these recipients, it is challenging to incorporate the knee implant (Moore et al., 2022). As a result, they experience sensations of disembodiment: they describe their knee as alien or ‘not their own’, lack a sense of connection (e.g. they occasionally trip if they do not think about the knee consciously), or do not trust that their knee could carry their weight (e.g. they are afraid to fall) (Moore et al., 2022). This case study shows that if implants are not successfully incorporated, this can disrupt (neutral) embodiment, which can result in poor treatment outcomes.

Regenerative valve implants could improve a person’s embodied experience, if symptoms related to heart valve disease are relieved. Yet, as the case study on knee implants shows, successful embodiment also requires that regenerative valve implants are incorporated into the body. Regenerative valve implants differ from inert knee implants, in that the smart lifelike material of the former becomes intimately interwoven with the tissues of the living body. We argued above that the implants become immersed in and entangled with the body on an ontological level also. We hypothesise that—at least for some recipients—this physical integration and ontological entanglement of implant and body might affect the incorporation process, and general state of embodiment, and this could be in two possible ways, as we explain below.

On the one hand, we hypothesise that the integration of the regenerative valve implant into the physical body might help some recipients to incorporate the implant into the body and to view the implant as part of them, as their own. The gradual disappearance of the implant and replacement by living tissue could moreover help to experience the implant as a normal part of the body, and foster body-self unity (neutral embodiment). This would be an improvement compared to existing valve implants, because the ‘inertness’ of mechanical valve implants (metal prosthetics), and the animal origin of biological valve implants (porcine transplants) could cause a sense of otherness (Moore et al., 2022) and contamination (Haddow, 2021), respectively, and could result in disembodiment. Moreover, the thudding sound of a mechanic valve implant closing with every heartbeat could cause undesirable body awareness (hyperembodiment) (Blome-Eberwein et al., 1996; Derksen & Horstman, 2008). In comparison, the degradation of the smart lifelike material and its replacement by living tissue could improve the recipient’s overall embodied experience, which would be beneficial for the therapeutic success of the treatment.

On the other hand, we hypothesise that some implant recipients might experience the integration of the regenerative valve implant into the living body as alienating, even if the treatment relieves disease symptoms. In addition to the surgical procedure—which could be experienced as a threat to bodily integrity (Lape et al., 2019)—knowing that the cells of the body enter the implant and the implant disintegrates and penetrates the body might be experienced as invasive and disruptive too. This could hinder incorporation of the implant into the body. Also the invisibility and disappearance of the implant could be disconcerting because, while intimately embedded inside the body, it is out of sight and out of control of the recipient and (close) others (Haddow et al., 2016). Thus, the entanglement of body and technology, living and non-living, might cause feelings of dissociation and discomfort and could result in disembodiment, which could negatively impact the therapeutic success of the treatment.

Ultimately, to understand what it means to be living with an implant requires an empirically informed account of the intimate relations between body and technology, and their embeddedness in a social context and a network of relations with others (Dalibert, 2016; Slatman, 2014). The question how recipients will incorporate regenerative valve implants and how this will affect their relation to their body and their embodied experience can therefore only be answered empirically after clinical implementation of the technology. We hypothesise that the process in which the implant integrates into the physical body and entangles with the ontological body might be soothing for some and discomforting for others. Prior qualitative studies have suggested that well-designed rehabilitation programmes can improve living with biological and mechanic heart valves (Hansen et al., 2018; Olsson et al., 2018; Oterhals et al., 2013). We therefore propose that, prior to surgery, a medical professional should discuss with the recipient how the treatment may affect their embodied experience. During the rehabilitation process, explicit attention should be paid to the process of incorporating the regenerative valve implant and to the recipient’s relationship with their body. Doing so may help recipients to deal with the surgical intervention and with living with a regenerative valve implant and may help them to perceive the implant as a supportive and welcome part of the body (Lape et al., 2019; Moore et al., 2022).

## Discussion

In our conceptual analysis, we have shown that, in the context of Regenerative Medicine, the notions ‘smart’ and ‘lifelike’ can be assigned specific meaning. ‘Smart’ refers to the two-way communication of materials with the tissues of the body through molecular signalling pathways. ‘Lifelike’ refers to the materials’ structural and functional similarity to, integration in, and interaction with the environment of living cells.

These meanings differ from their use in other smart and biomimetic technologies, as summarised in Table 1. In smart information technologies, ‘smart’ indicates the collection and exchange of digital or electronic data (Haddow et al., 2016). While in both cases smart refers to a feedback loop of sensing and responsiveness, the means of communication differs. The notion ‘lifelike’ in regenerative valve implants closely resembles that of ‘biomimicry’ in the context of sustainable and ecological design (Blok, 2023). Yet lifelike materials are more intimately interwoven with their living environment, i.e. the human body.

In the context of Regenerative Medicine, it seems likely that new materials will be developed that are ‘smarter’ and ‘more lifelike’ than the ones currently available. Research into new types of materials in the laboratory moves much faster than the translation of regenerative valve implants to the clinic. By the time the implants will be clinically available, most likely a next generation of materials will already be under development. Because of the ongoing convergence between nanotechnology, biotechnology, information technology, and cognitive science (NBIC) (Van Est, Stemerding, et al., 2014), these materials can be expected to show emergent properties, i.e. new forms of smartness and lifelikeness, that differ from what we described so far. Our conceptual analysis of smart lifelike materials above allows for a preliminary exploration of how smart lifelike materials might develop in the future.

Regarding future forms of ‘smartness’, a convergence could take place with a *different* type of smart technologies, namely wearable or implantable information technologies such as smart watches and smart pills. While speculative, this is not unconceivable because nanotechnology is the driving force behind both types of smartness (Raman & Bashir, 2017; Van Est, Rerimassie, et al., 2014; Van Est, Stemerding, et al., 2014; Walhout et al., 2010; Wu et al., 2017). Materials might be developed that combine the smartness of regenerative implants (i.e. the capacity to sense and respond to living tissues through molecular signalling pathways) with the smartness of wearable or implantable information technologies (i.e. the capacity to collect information about the body, and store, process and/or exchange it digitally with a device outside the body, and to respond to this information accordingly) (Haddow et al., 2016). This next generation of smart materials could be applied in the design of regenerative implants that could stimulate tissue regeneration *and* collect health data about the human body (Kim et al., 2022). Possibly these data could even be used to provide feedback on how tissue regeneration progresses and adjust the process if necessary.

Moreover, with regards to future forms of ‘lifelikeness’, it might become possible to mimic not only the structure and function of the extracellular matrix, but also that of living cells themselves. Such cell mimics are called ‘synthetic cells’ or ‘artificial cells’. Several research groups worldwide are attempting to build synthetic cells bottom-up, by assembling macromolecular elements in a lipid encapsulation to create entities with—ultimately—self-reproducing capacities (Buddingh’ & Van Hest, 2017; Powell, 2018). Conceivably, once successful, synthetic cells could be assembled into synthetic tissues (Bayley et al., 2019; Stevendaal et al., 2021). Thus, a next generation of lifelike materials could emerge that is synthetically derived but has near living characteristics, and therefore mimics tissues even more closely. Applied to tissue regeneration (Hu et al., 2018), in theory this could enable the design of regenerative implants with improved signalling and responsiveness to bodily tissues and enhanced regenerative capacities.

Such future forms of smartness and lifelikeness would, if actualised, provide opportunities, but also present additional ethical challenges. Here we focus specifically on questions about control. Future smart lifelike materials could improve control over bodily processes of tissue regeneration and thereby restore a diseased body to a state of physical health that would not otherwise be possible. Yet increased control also comes with increased risk, especially if control is lost. For example, future smart materials capable of automated real-time health data measurements would be out of reach of both physician oversight and recipient control, and could pose a threat to device security and data privacy if control over data access is lost (Haddow et al., 2016). Future lifelike materials, moreover, could acquire emergent properties that are hard to predict or control, and that could therefore pose a threat to human health or the natural environment (Bedau et al., 2009). Built-in safeguards in order to control data access and to control lifetime and performance (Raman & Bashir, 2017) would therefore be desirable. A broader concern might also be raised regarding limits to legitimate control over living systems (Habets et al., 2021; Maienschein, 2009). To guarantee continued responsible development, the emergent properties and ethical implications of future smart lifelike materials should be anticipated and analysed in more detail early on, for example by means of normative hermeneutic approaches to established methods in future studies (Brey, 2012; Grunwald, 2014).

## Conclusion

In this article, we explored the conceptual and ethical implications of the development of smart lifelike materials for the design of regenerative implants, using heart valve implants as a showcase. We showed that the materials are considered ‘smart’ because they can communicate with human tissues, and ‘lifelike’ because they are structurally similar to these tissues. From this conceptual understanding of smart lifelike materials we drew insights on the ontological status of regenerative valve implants, which in turn informed our ethical analysis. We showed that the integration of the implants with the tissue of the body makes the implantation procedure highly irreversible, which has implications for risks, responsibility and control. Moreover, we hypothesised that the integration and eventual disappearance of the implants inside the body might promote embodiment for some but might cause alienation and disembodiment for others. While these challenges could arise in other domains of Regenerative Medicine too, they become exacerbated in the case of smart, lifelike materials and regenerative valve implants due to the intimate intertwinement of the implants with the tissues of the human body, and the resulting ontological entanglement of body and technology. In our discussion, we preliminarily explored what new forms of smartness and lifelikeness might be developed in the future, how that may change the design of regenerative implants, and what new ethical challenges that would give rise to.

With our analysis, we hope to contribute to responsible development of smart lifelike materials and regenerative implants. We recommend that further research efforts be undertaken to anticipate and evaluate future developments in the field of smart lifelike materials more in depth. Moreover, the ethical challenges related to irreversibility and embodiment posed by regenerative valve implants in their current design should be actively accounted for during technological development, as well as during clinical translation and implementation. Doing so will ensure that regenerative valve implants will not merely regenerate valve tissue and restore the pumping function of the heart but will truly improve the everyday life and lived experience of the recipient.

## Footnotes

1 NB: Tissue engineers usually refer to synthetic materials used for implantation in a human body as ‘biomaterials’. The prefix ‘bio-’ refers to the compatibility of the non-living material with a living system, e.g. the human body (“The Glossary for Cell & Gene Therapy and Regenerative Medicine 2020,” 2020). In this paper we prefer to use the bare term ‘material’ in order to prevent confusion with ‘bodily material’, i.e. living human cells and tissues, which are sometimes (though less conventionally) also referred to as biomaterials.

2 Mechanical valve implants (made from metal and other inert materials) require anticoagulation medication (blood thinners), which may cause bleeding (Nishimura & Warnes, 2015). And biological valve implants (i.e. xenografts, usually made from decellularized porcine valve tissue) have a limited lifetime and therefore do not provide a sustainable solution for younger and middle aged people with heart valve disease (Manji et al., 2015; Mol et al., 2009).

3 See footnote 1.

4 For example, experiments in animal models have shown that sometimes implanted materials lose their ‘smart’ properties within days after implantation because they are modified by bodily processes like the blood flow and immune response (Uiterwijk et al., 2020). This indicates that the biological processes in the body can overrule the intended scheme of the engineer.
